# A Waveform Image Method for Discriminating Micro-Seismic Events and Blasts in Underground Mines

**DOI:** 10.3390/s20154322

**Published:** 2020-08-03

**Authors:** Hui Wei, Weiwei Shu, Longjun Dong, Zhongying Huang, Daoyuan Sun

**Affiliations:** 1 School of Traffic & Transportation Engineering, Changsha University of Science & Technology, Changsha 410114, China; wh@csust.edu.cn; 2 School of Resources and Safety Engineering, Central South University, Changsha 410083, China; lj.dong@csu.edu.cn (L.D.); sundaoyuan@csu.edu.cn (D.S.); 3 School of Mathematics, Hunan University, Changsha 410082, China; norahuang@hnu.edu.cn

**Keywords:** micro-seismic event, blast, discrimination, waveform image, underground mines

## Abstract

The discrimination of micro-seismic events (events) and blasts is significant for monitoring and analyzing micro-seismicity in underground mines. To eliminate the negative effects of conventional discrimination methods, a waveform image discriminant method was proposed. Principal component analysis (PCA) was applied to extract the raw features of events and blasts through their waveform images that established by the recorded field data, and transform them into the new uncorrelated features. The amount of initial information retained in the derived features could be determined quantitatively by the contribution rate. The binary classification models were established by utilizing the support vector machine (SVM) algorithm and the PCA derived waveform image features. Results of four groups of cross validation show that the optimal values for the accuracy of events and blasts, total accuracy, and quality evaluation parameter MCC are 97.1%, 93.8%, 93.60%, and 0.8723, respectively. Moreover, the computation efficiency per accuracy (CEA) was introduced to quantitatively evaluate the effects of contribution rate on classification accuracy and computation efficiency. The optimal contribution rate was determined to be 0.90. The waveform image discriminant method can automatically classify events and blasts in underground mines, ensuring the efficient establishment of high-quality micro-seismic databases and providing adequate data for the subsequent seismicity analysis.

## 1. Introduction

Micro-seismic monitoring has been effective in global underground mines for providing information about the local state of stress of rock [1]. It also provides information that can be used to understand the behavior of rock mass [2,3], to prevent rock mass instability and rock burst [4,5], and to assess the potential hazards induced by intensive deep mining activities [6,7]. The discrimination of micro-seismic events and blasts in underground mines is one of the most important issues for the robust and efficient micro-seismic monitoring technology [8], which directly affects the quality of micro-seismic database [9,10]. The reasonable and reliable interpretation of underground process of mining can be obtained only by using the pure data of micro-seismic events rather than the mixed data of micro-seismic events and blasts [11]. Otherwise, the location accuracy of micro-seismic events will be reduced seriously [12]. Moreover, the resolution of passive tomography will be influenced negatively because the accurate localization of micro-seismic events is necessarily a basis. Subsequently, the related analysis of rock deformation and stress evolution may be ineffective. Therefore, the false discrimination of micro-seismic events and blasts may result in the unreasonable assessment of seismic hazards, such as a fictitious region of high seismic stress [13]. It is evident that the discrimination of micro-seismic events and blasts in underground mines is a fundamental and significant problem to be solved.

The manual discrimination has been widely used to classify micro-seismic events and blasts based on the blast time and the visual judgment of waveforms. However, it is common that a great deal of monitoring data needs to be processed routinely, which will result in a long discrimination time, low work efficiency, and delayed classification results. Also, the professional knowledge and practical experience are necessary for the data analysts, whose technical levels will have an impact on the classification results. It can be seen that the manual discrimination is inefficient and leads to difficulties for fast analysis of seismic sources, determination of rock mass conditions, and assessment of seismic hazards in underground mines.

To improve the classification efficiency, numerous discrimination methods have been proposed to automatically discriminate micro-seismic events and blasts in underground mines by using various parameters derived from seismic source and waveform characteristics. These methods can mainly be classified into three groups, which are the waveform spectrum analysis [14,15,16,17,18,19,20,21], the statistical methods such as the maximum-likelihood Gaussian classifier [22,23] and the linear discrimination methods [24,25], the machine learning methods including neural networks [26,27,28,29], self-organizing map [30,31], diffusion maps [32], etc.

Different seismic source parameters are selected to establish discrimination models for micro-seismic events and blasts by using statistical methods. Malovichko [33] applied the maximum-likelihood Gaussian classifier to discriminate micro-seismic events and blasts, for which the source parameters including time of occurrence, radiation pattern, ratio of high- and low-frequency radiation, and source repetition to the neighboring waveforms were selected as indicators. Vallejos and McKinnon [34] selected 13 seismic source parameters provided by the full-waveform system. Through the comparison of classification accuracy, they found that the neural network models outperformed the current approach, indicating good classification performance of machine learning methods. Based on the seismic source parameters proposed in Dong et al. [13], Dong et al. [35] applied the logistic and log-logistic distributions to establish probability density functions for origin time of blasts and origin time difference (OTD) of neighboring blasts in the time domain. Then, the Fisher classifier, naive Bayesian classifier, and logistic regression were used to establish discrimination models with explicit functions. However, the values of seismic source parameters may be unstable with the change of source coordinates, wave velocity, and location error. In summary, the discrimination methods using seismic source parameters mainly have four prominent disadvantages that may lead to poor discrimination results. Firstly, there are dozens of initial seismic source parameters in mine seismicity, which mainly includes location error (*E_rr_*), origin time of seismic records (*t*_0_), source radius (*R*), number of triggered sensors (*N_s_*), moment magnitude (*M_m_*), seismic moment (*M*_0_), total radiated energy (*E*_0_), corner frequency (*f_c_*), maximum displacement (MD), peak velocity parameter (PV), etc. There will be more derived parameters definitely by applying various functions, as well as by combining and transforming different variables. The workload for analyzing each initial and derived parameter will be heavy as the statistical analysis is the most common method for selecting parameters with good discrimination performance. Secondly, the combinations of acceptable parameters corresponding to different discrimination models are various and complex. Thirdly, the importance of each parameter is usually ignored without determining a quantitative value and the established classification models lack the process for eliminating the correlations between different parameters. Fourthly, the selection of seismic source parameters and distribution functions are sometimes carried out with experience and subjective judgment.

As for the waveform spectrum analysis, its advantage lies in that different characteristics between micro-seismic events and blasts can be analyzed intuitively [15]. Nevertheless, its analysis object is the waveform recorded by each sensor, the workload is heavy as numerous sensors will be triggered by a micro-seismic event or a blast. Zhao et al. [36] selected the repetition of waveforms, tail decreasing, dominant frequency, and occurrence time as the discrimination indicators. Besides, for micro-seismic events and blasts, by considering the differences of the time needed to reach the first and the main peak and the amplitude distribution, the slope values of two regression lines (one corresponds to the first peak while another corresponds to the main peak) were extracted as the characteristic parameters for waveforms. Ma et al. [37] proposed two discrimination approaches, where one extracted features from seismic sources (Approach I) and another utilized waveform characteristics (Approach II). The results showed that 97.1% of cases were correctly classified by Approach II while the accuracy of Approach I was only 83.5%. According to both two researches, it can be inferred that waveform characteristics can provide useful information for effective discrimination. Hence, three main disadvantages of waveform spectrum analysis can be concluded through the above review. Firstly, the workload of waveform spectrum analysis is heavy, because the analysis object is the waveform recorded by each sensor and many sensors will be triggered by a single micro-seismic event or a blast. Secondly, in general, only the characteristics of the P-wave are considered, instead of the full waveform. Thirdly, the importance of each parameter is usually ignored without determining a quantitative value and the established classification models lack the process for eliminating the correlations between different parameters, which is also an identical disadvantage of many discrimination methods using seismic source parameters.

In this paper, we developed an effective waveform image method for discriminating micro-seismic events and blasts in underground mines. Firstly, we established the waveform image databases of micro-seismic events and blasts through the full waveform data. Then, we used PCA [38,39,40] to extract the original image features and obtained the new uncorrelated features with quantitative importance and lower dimensions. Thirdly, we developed the discrimination models by utilizing the support vector machine (SVM) algorithm [41,42,43] and the PCA derived features. Finally, we analyzed the discrimination results of cross validations, quantitatively evaluated the effects of contribution rate on classification accuracy and computation efficiency, and discussed the further application, advantages, and disadvantages of the proposed discriminant method.

## 2. Materials and Methods

**Figure 1:**
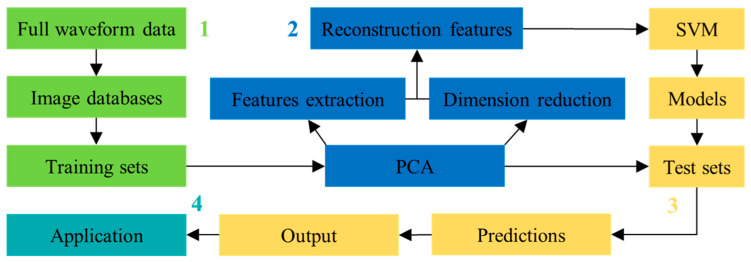
Main steps of the proposed waveform image discriminant method. The green, blue, yellow, and cyan squares represent steps 1, 2, 3, and 4, respectively. 1. Establishment of waveform image databases; 2. Principal component analysis; 3. Establishment of discrimination models; 4. Application of the discrimination results.

Figure 1 illustrates the proposed waveform image method for discriminating micro-seismic events in underground mines, which are mainly divided into four steps.

1. Establishment of waveform image databases: The full waveform data recorded from 2013 to 2015 by a micro-seismic monitoring system installed in the Yongshaba underground mine is used to produce the waveform images. Then, the waveform image databases of micro-seismic events and blasts are established, where micro-seismic events and blasts are labeled as E and B, respectively.
2. Principal component analysis: PCA is applied to extract the original image features from the two databases and transform them into the new uncorrelated features with quantitative importance and lower dimension, where the amount of initial information retained in the derived features is determined by the contribution rate. Thus, PCA can reduce the number of input features and improve the classification efficiency.
3. Establishment of discrimination models: SVM algorithm is selected to establish discrimination models for micro-seismic events and blasts in underground mines by utilizing the PCA derived waveform image features. Then, the discrimination models are used to classify for test sets.
4. Application of the discrimination results: The micro-seismic data is applied to locate micro-seismic events, to analyze the local state of stress of rock, and to assess potential hazards in underground mining area.

### 2.1. Establishment of Waveform Image Databases

The generation of waveforms, the determination of a reasonable and unified signal duration for all the waveforms, as well as the generation and definition of waveform images are the key issues for establishing waveform image databases of micro-seismic events and blasts.

Firstly, the micro-seismic monitoring system installed in the Yongshaba underground mine is composed of 28 sensors that measure the ground velocity and their sampling frequency is 6000 Hz, which means that the ground velocity is measured for 6000 times during one second. Hence, the waveforms of micro-seismic events and blasts can be produced through the data of time and the corresponding ground velocity, where the *x*-axis and *y*-axis represent waveform time (*s*) and velocity amplitude (*m*/*s*), respectively.

**Figure 2:**
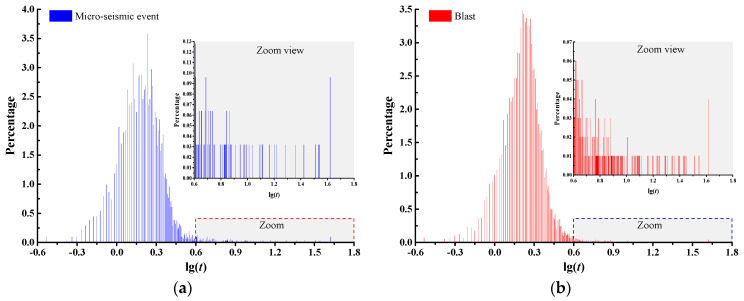
Distributions and percentages of different signal duration for micro-seismic events and blasts. (**a**) Distributions and percentages of signal duration for micro-seismic events; (**b**) distributions and percentages of signal duration for blasts.

Secondly, the signal durations of a micro-seismic event and a blast are supposed to present differences due to the differences of their energy release [36]. Here, we quantify the distributions and percentages of different signal durations for micro-seismic events and blasts, which can subsequently be used to determine a reasonable and unified signal duration for all the waveforms. Figure 2 shows the distributions and percentages of lg(*t*) for micro-seismic events and blasts, where *t* denotes the signal duration.

It can be seen that there are numerous different values of signal duration for the waveforms of micro-seismic events and blasts, which account for different percentages. Therefore, it is necessary to determine a reasonable and unified signal duration for all waveforms, which can avoid the changes of image features and inaccuracy of subsequent discrimination models caused by different signal duration of micro-seismic events and blasts. *t_ei_* (*i* = 1, 2,…, *n_e_*) and *t_bj_* (*j* = 1, 2,…, *n_b_*) denote the signal duration of the *i*-th micro-seismic event and *j*-th blast, respectively. *n_e_* and *n_b_* indicate the total numbers of micro-seismic events and blasts, respectively. The percentages of each signal duration of micro-seismic events and blasts are calculated as

(1) $$\left\{ \begin{array}{l} \eta_{p}=\frac{n_{t_{ei}}}{n_{e}}\\ \xi_{q}=\frac{n_{t_{bi}}}{n_{b}} \end{array}\right.,$$

where $n_{t_{ei}}$ denotes the numbers of micro-seismic waveforms whose signal duration are equal to *t_ei_* and $n_{t_{bi}}$ denotes the numbers of blast waveforms whose signal duration are equal to *t_bj_*. *η_p_* (*p* = 1, 2,…, *l*,…, *x*) indicates the percentages of different micro-seismic signal durations (i.e., *t_ei_*) in all micro-seismic events and *ξ_q_* (*q* = 1, 2,…, *k,*…, *y*) indicates the percentages of different blast signal durations (i.e., *t_bj_*) in all blasts. By sorting the resolved percentages with the descending order, thus, *η*_1_ and *η_x_* correspond to the micro-seismic signal durations that have the maximum number (percentage) and the minimum number (percentage), respectively. Then, by setting a threshold of 80% for the sum of percentages of micro-seismic signal durations (or blast signal durations), the reasonable and unified signal duration of micro-seismic events and blasts can be solved through Equations (2)–(4)

(2) $$\left\{ \begin{array}{l} \eta_{1}+\eta_{2}+\cdots +\eta_{l}\geq 80\%\\ \xi_{1}+\xi_{2}+\cdots +\xi_{k}\geq 80\% \end{array}\right.,$$

(3) $$\left\{ \begin{array}{l} t_{e}=\frac{t_{e1}+t_{e2}+\cdots +t_{el}}{l}\\ t_{b}=\frac{t_{b1}+t_{b2}+\cdots +t_{bk}}{k} \end{array}\right.,$$

(4) $$t_{s}=ceil\left[\max\left(t_{e},t_{b}\right)\right],$$

where *l* and *k* are the critical values that make the Equation (2) true. *t_e_* and *t_b_* indicate the average signal duration of micro-seismic events and blasts, respectively. The function *ceil* means rounding upward the first decimal place of the maximum value between *t_e_* and *t_b_*. *t_s_* denotes the reasonable and unified signal duration of micro-seismic events and blasts that eliminated the effects of extreme values.

In this study, the values of *t_e_*, *t_b_*, and *t_s_* are equal to 1.71 s, 1.63 s, 1.80 s, respectively. The signal duration of 1.8 s is also consistent with the percentage distributions in Figure 2, where the percentages around 0.25 (lg1.8) are relatively higher than others. It can be concluded that the signal duration of 1.8 s is reasonable for the establishment of waveform image databases and further classification study. As for the waveforms with signal duration greater than 1.8 s, only the part within 1.8 s is selected and the extra part is aborted. As for the waveforms with signal duration less than 1.8 s, the *y*-values of the part that ranges from the actual tail to 1.8 s are set to be 0.

**Figure 3:**
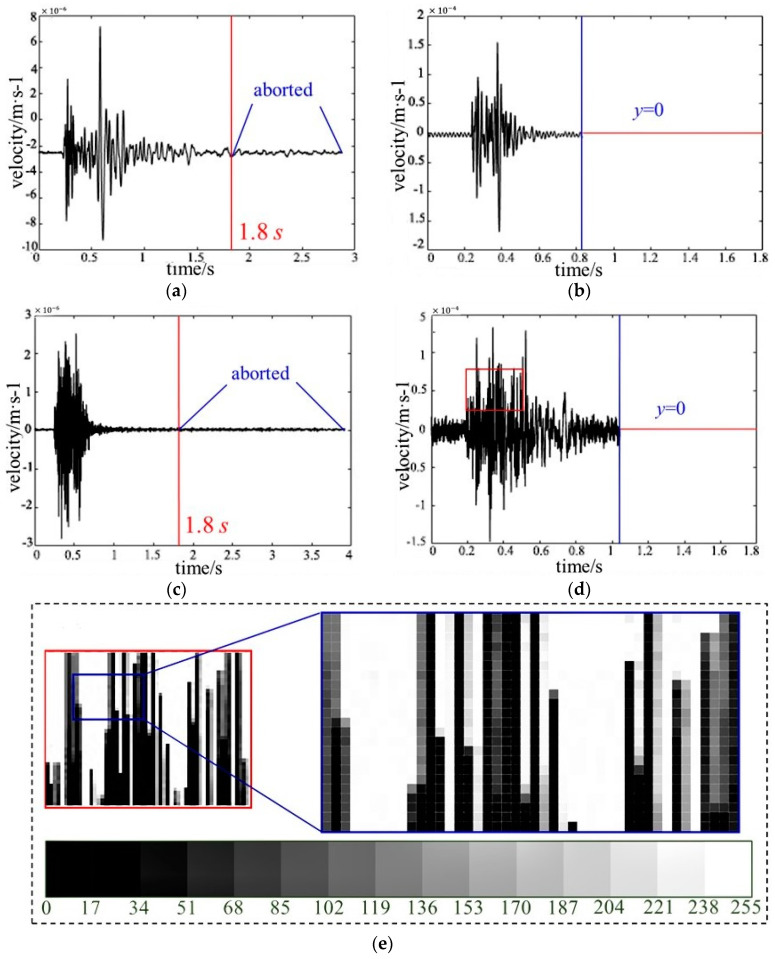
Examples of waveform images of micro-seismic events and blasts. (**a**) Waveform image of a micro-seismic event whose signal duration is greater than 1.8 s; (**b**) Waveform image of another micro-seismic event whose signal duration is less than 1.8 s; (**c**) Waveform image of a blast whose signal duration is greater than 1.8 s; (**d**) Waveform image of another blast whose signal duration is less than 1.8 s; (**e**) A part of pixels distributed in the red rectangle of (**d**), which are surrounded by the blue rectangle, and the corresponding gray values. The pure black and pure white correspond to the gray values of 0 and 255, respectively.

Thirdly, the waveforms of micro-seismic events or blasts with a unified signal duration (1.8 s) generated through the previous steps are saved as images (.png) with a unified resolution and size, named waveform images, where the unified resolution and size can ensure the number of the original waveform image features of all the waveforms is equal. To reduce the memory usage in the subsequent computational process, the resolution is set to be 400 × 300. Figure 3 shows four examples of waveform images of micro-seismic events and blasts, as well as a part of pixels of a waveform image and the corresponding gray values. Subsequently, the features of each waveform image can be extracted and represented by the gray values between 0 and 255 that corresponds to different pixels.

### 2.2. Principal Component Analysis

The database of micro-seismic events is taken as an example to clarify the main theory of PCA [38,39,40]. We can extract the original features from the micro-seismic database and present them by a 2D matrix $X_{mn}$, which consists of *m* row vectors ($x_{1n},x_{2n},\cdots ,x_{mn}$). *m* and *n* denote the number of waveform images and the number of original features extracted from each waveform image, respectively. To eliminate the errors caused by the original features with different scales, the Min-Max method is selected to normalize the original features, which is explained below

(5) $${x^{\prime }}_{ij}=\frac{x_{ij}-\min}{\max-\min},$$

where $x_{ij}$, ${x^{\prime }}_{ij}$, min, and max are the initial *j*th feature value of the *i*th waveform image, the normalized *j*th feature value of the *i*th waveform image, the minimum feature value, and the maximum feature value, respectively. Then, the new original features of seismic database are presented by the normalized matrix ${X^{\prime }}_{mn}$, which consists of *m* normalized row vectors (${x^{\prime }}_{1n},{x^{\prime }}_{2n},\cdots ,{x^{\prime }}_{mn}$). Furthermore, the difference matrix $X_{d}$, which can retain differences between different waveform images, and its covariance matrix $C_{nn}$ as well as the dimension reduction matrix $X_{\mathrm{DR}}$ are solved as

(6) $$X_{d}={X^{\prime }}_{mn}-{\left(X_{\mathrm{ave}}\right)}_{m\times n},$$

(7) $$w\left(\lambda_{1}\right)+w\left(\lambda_{2}\right)+\cdots +w\left(\lambda_{k}\right)\geq \sigma ,$$

(8) $$X_{\mathrm{DR}}=\left[\begin{array}{c} e_{1k}\\ e_{2k}\\ \vdots \\ e_{nk} \end{array}\right]=\left[\begin{array}{l} e_{11},e_{12},\cdots ,e_{1k}\\ e_{21},e_{22},\cdots ,e_{2k}\\ \begin{array}{c} \vdots \end{array}\begin{array}{c} \begin{array}{c} \begin{array}{c} \vdots \end{array} \end{array}\ddots \vdots \end{array}\\ e_{n1},e_{n2},\cdots ,e_{nk} \end{array}\right],$$

where $X_{\mathrm{ave}}$ consists of *m* identical row vectors that are composed of the average value of each column in the matrix ${X^{\prime }}_{mn}$. $\lambda_{j}$ are the eigenvalues of $C_{nn}$ that sorted by the descending order. **e***_j_* and $w\left(\lambda_{j}\right)$ are the eigenvectors and importance that correspond to $\lambda_{j}$, respectively. *σ (0 ≤ σ ≤ 1)* is the contribution rate that quantitatively determines the amount of initial information retained in the PCA derived features. *k* (*k ≤ n*) is the smallest integer that satisfies the preset contribution rate *σ*. Finally, the new uncorrelated features with quantitative importance and lower dimension, named principle components (PCs), can be presented by a reconstruction matrix $X_{R}$, which is calculated as

(9) $$X_{R}={X^{\prime }}_{mn}\cdot X_{\mathrm{DR}},$$

Therefore, PCA can be utilized to objectively extract the original waveform image features from the databases of micro-seismic events and blasts, as well as to transform them into the new uncorrelated features, which can be used for establishing discrimination models.

### 2.3. Classification Algorithm

SVM algorithm, proposed by Cortes and Vapnik [41], has indicated excellent performances in the fields of regression, classification, and pattern recognition. As for binary classification problems, the basic thought of SVM algorithm is to search an optimal hyperplane between two objects that can maximize the margin area while ensuring the classification accuracy. The flexibility allows us to modify and improve the SVM algorithm, as well as to conveniently apply it to different situations according to the specific requirements. In addition, the number of initial input parameters can be decreased by providing more default parameters, which can reduce the work for parameters adjustment and accelerate the computation process.

Therefore, SVM algorithm is used to establish discrimination models for micro-seismic events and blasts by using the training samples. Then, the effectiveness of classification models will be examined through the test samples.

### 2.4. Evaluation of Classification Quality

The Matthews correlation coefficient (MCC), proposed by Matthews [44], is a commonly used index in machine learning for evaluating binary classification quality, which can simultaneously consider the classification accuracy of micro-seismic events and blasts. Essentially, MCC is a correlation coefficient between the observed and the predicted binary classifications. The value interval of MCC is [−1, 1], where −1 represents total falseness between observation and predication, 0 denotes no better than random prediction, and 1 indicates absolute correctness for prediction. MCC is defined and calculated as

(10) $$\mathrm{MCC}=\frac{\mathrm{TE}\times \mathrm{TB}-\mathrm{FE}\times \mathrm{FB}}{\sqrt{\left(\mathrm{TE}+\mathrm{FE}\right)\left(\mathrm{TE}+\mathrm{FB}\right)\left(\mathrm{TB}+\mathrm{FE}\right)\left(\mathrm{TB}+\mathrm{FB}\right)}},$$

where a true micro-seismic event (TE) means that a micro-seismic event is identified as a micro-seismic event, a true blast (TB) means that a blast is identified as a blast, a false micro-seismic event (FE) means that a blast is incorrectly tagged as a micro-seismic event, and a false blast (FB) means that a micro-seismic event is incorrectly tagged as a blast.

## 3. Experimental Study and Results

### 3.1. Data Description and Preparation

**Figure 4:**
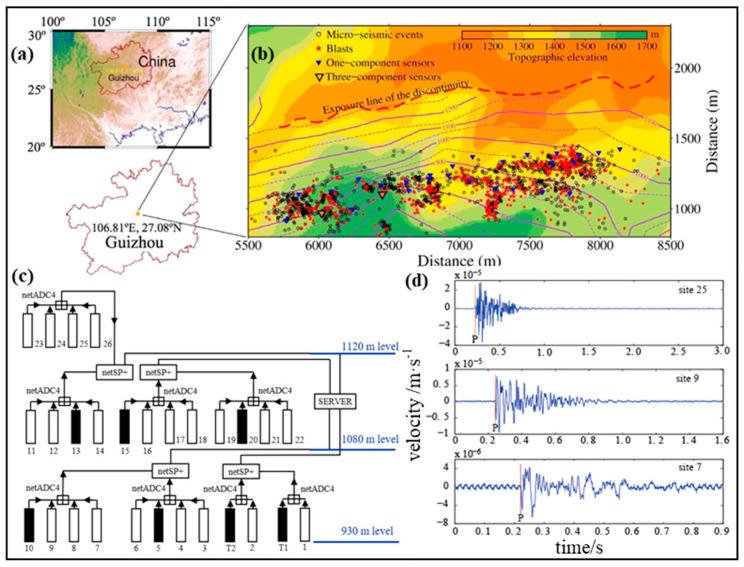
Map view of the Yongshaba underground mine. (**a**) Geographic location of the Yongshaba underground mine; (**b**) Mine structure as well as the locations of micro-seismic events, blasts and the sensors; (**c**) Layout of the uniaxial velocity sensors (from 1 to 26) and the triaxial velocity sensors (T1 and T2); (**d**) Examples of recorded waveforms.

The full waveform data of micro-seismic events and blasts recorded from 2013 to 2015 by the Institute of Mine Seismology (IMS) system installed in the Yongshaba deposit, an underground mine in Guizhou Province, China, was used to establish databases and discrimination models. Twenty-six uniaxial sensors and two triaxial sensors, measuring the ground velocity with a sampling frequency of 6000 Hz, were deployed across the major stopes at the 930 m level, 1080 m level and 1120 m level, which can cover the main mining area and record the data of mining-induced seismicity and production blasts as much as possible. Figure 4 shows the geographic location of the Yongshaba underground mine, the locations of micro-seismic events and blasts [45], the layout of the sensors, and the examples of waveforms recorded by different sensors.

To ensure the generality for different micro-seismic data of the proposed discriminant method, 2000 micro-seismic events and 2000 blasts are randomly selected from the established waveform image databases. The cross validation, an effective method for evaluating discrimination models, is used in this study, whose basic thought is establishing discrimination models through the training sets (test sets) and evaluating the established models through test sets (training sets). Therefore, 2000 micro-seismic events are equally divided into E1 and E2, and 2000 blasts are equally divided into B1 and B2, where E1 and E2 indicate the first and the second micro-seismic dataset containing 1000 micro-seismic events, respectively, and B1 and B2 represent the first and the second blasting dataset consisting of 1000 blasts, respectively. Thus, four groups of cross validation can be carried out through the combinations of these four datasets (E1, E2, B1, and B2), which are shown in Table 1.

### 3.2. PCA Application and Analysis

PCA is applied to the four groups of cross validation, where the contribution rate is firstly set to be 95% as it is a commonly used value that has shown good classification performances in many fields such as the facial recognition. Hence, the first 95% information contained in the original waveform image features is retained in the PCA derived waveform image features. The eigenvalues, importance, and cumulative importance corresponding to different PCs for test 1 to test 4 are listed in Table 2.

*D*_orig_ and *D*_redu_ indicate the dimension of the original features and the derived features, respectively. *D*_orig_ cannot be greater than 2000, which is the capacity of each training set. PC*_D_*_redu_ is the final principle component that satisfies the cumulative importance of 0.95. With the increase of principle components, their eigenvalues and importance tend to decrease. As for test 1 to test 4, all the dimensions of the original features are 2000, while the dimensions of the derived features are reduced to 1157, 1166, 1195, and 1205, respectively. Additionally, it can be calculated that the average importance of PC1 for test 1 to test 4 is about 8.68% [(9.20% + 9.67% + 7.73% + 8.11%)/4 = 8.68%], which is 173.6 times as large as the average importance of an original feature (1/2000 = 0.05%).

**Figure 5:**
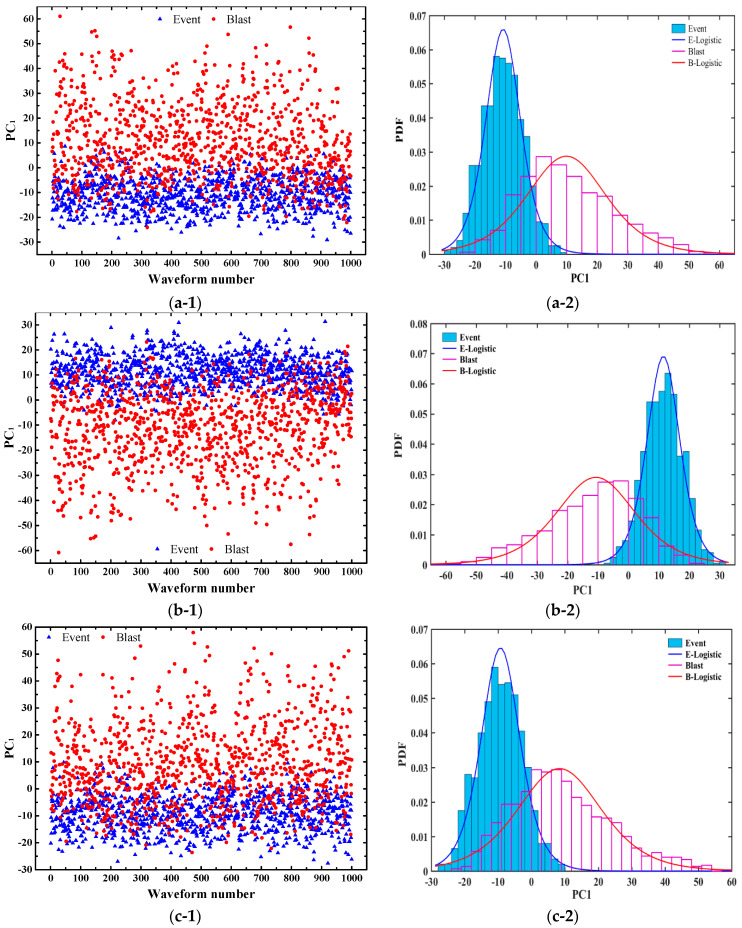

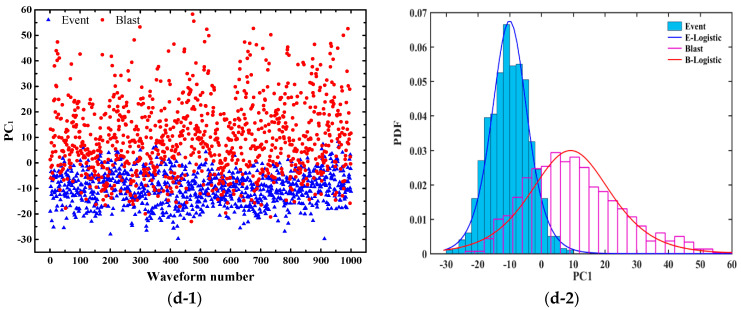
Distributions and the logistic probability density distributions of PC_1_ of micro-seismic events and blasts for test 1 to test 4. (**a**) Test 1; (**b**) Test 2; (**c**) Test 3; (**d**) Test 4. The blue triangles and red circles indicate the micro-seismic events and blasts in the training sets, respectively.

Figure 5 shows the distributions and the logistic probability density distributions of PC_1_ of micro-seismic events and blasts for test 1 to test 4. It can be seen from the left figures that the differences between micro-seismic events and blasts are evident. Also, the overlapped areas between micro-seismic events and blasts under the logistic probability density distributions in the right figures are small. Therefore, the effectiveness of PC_1_ for discriminating micro-seismic events and blasts is confirmed and we can believe that the PCA derived waveform image features are effective and efficient for the further discrimination of micro-seismic events and blasts.

**Figure 6:**
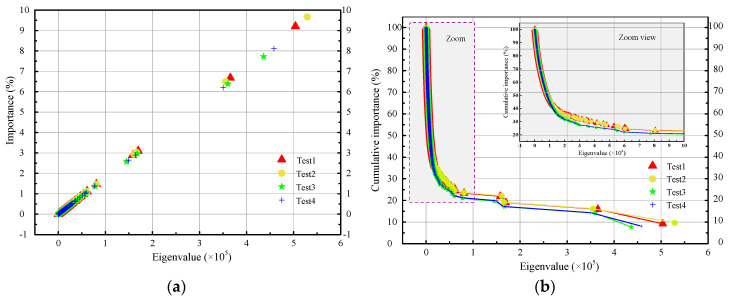
Importance and cumulative importance of the PCA derived eigenvalues for test 1 to test 4. (**a**) Importance of the PCA derived eigenvalues for test 1 to test 4. The red triangles, yellow circles, green stars, and blue crosses indicate the eigenvalues with specific importance for test 1 to test 4, respectively; (**b**) Cumulative importance of the PCA derived eigenvalues for test 1 to test 4. The red line with triangles, yellow line with circles, green line with stars, and blue line with crosses represent the cumulative importance curves for test 1 to test 4, respectively. The zoom view shows the cumulative importance curves when the eigenvalue is between 1 × 10^4^ and 10 × 10^4^.

Figure 6 shows the importance and cumulative importance of the PCA derived eigenvalues for test 1 to test 4. It can be seen that the importance decreases and the cumulative importance increase with the reduction of eigenvalues. In Figure 6, the eigenvalues with smaller importance are distributed at the lower left corner. For the cumulative importance curves of test 1 to test 4, the upper left parts are constituted of numerous eigenvalues with relatively smaller importance. The PCA derived waveform image features that contribute the first 95% cumulative importance are the input features for the establishment of discrimination models.

### 3.3. Classification Results

Usually, the radical basis function (RBF) is used as the kernel function of SVM algorithm for its good performance in common classification problems. However, RBF is not suitable when the dimension of the input features is very large, while the linear kernel function is an advisable choice considering the greater dimension of input features in further engineering applications. In addition, there is no need to set or adjust numerous parameters for linear kernel function and the classification process can be simplified. Therefore, the linear kernel function is selected for SVM algorithm, which is given by

(11) $$K\left(E,B\right)=B^{T}\cdot E,$$

where **E** and **B** indicate the training sets of micro-seismic events and blasts, respectively.

By inputting the PCA derived waveform image features, the discrimination models can be established through SVM algorithm. The classification results for test 1 to test 4 including classification accuracy and quality evaluation factor MCC are shown in Table 3, where TE and TB are the numbers of correctly classified micro-seismic events and blasts.

The classification accuracy of micro-seismic events for test 1 to test 4 are 95.0%, 92.3%, 97.1%, and 94.7%, respectively. Similarly, the discriminant accuracy of blasts for test 1 to test 4 are 92.2%, 93.8%, 89.9%, and 89.0%, respectively. The average classification accuracy of micro-seismic events and blasts for test 1 to test 4 are 94.78% and 91.23%, respectively. It can be seen that the optimal classification accuracy of micro-seismic events and blasts are 97.1% and 93.8%, respectively. All the four tests show excellent total accuracy, where the greatest value, the smallest value, and the average value are 93.60%, 91.85%, and 93.00%, respectively. The average value and the optimal value of MCC are 0.8607 and 0.8723, respectively, which are close to the upper limit of its value interval [−1,1]. The classification results indicate that the proposed waveform image method has excellent discriminating performance in underground mines.

## 4. Discussion

### 4.1. Contribution Rate

**Figure 7:**
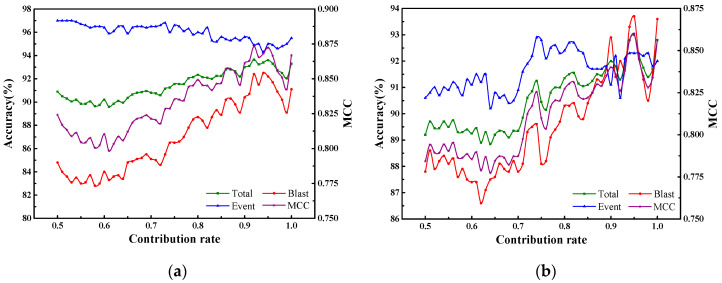

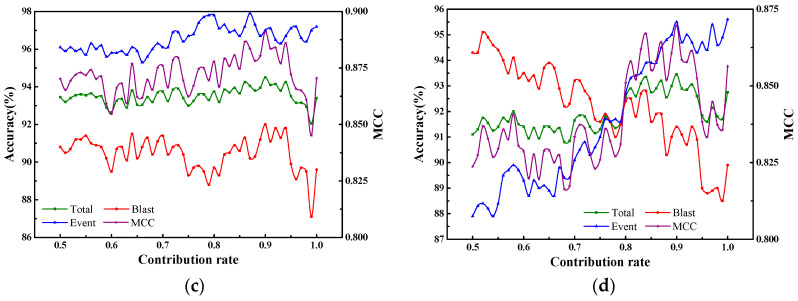
Classification accuracy of micro-seismic events and blasts, total classification accuracy, and quality evaluation parameter MCC for test 1 to test 4 under different contribution rates. (**a**) Test 1; (**b**) Test 2; (**c**) Test 3; (**d**) Test 4. The blue line with triangles, red line with circles, green line with squares, and purple line with stars indicate the classification accuracy of micro-seismic events, classification accuracy of blasts, total accuracy, and MCC, respectively.

The contribution rate is a key parameter that quantitatively determines the amount of initial information retained in the input features derived from PCA. An appropriate value of the contribution rate can not only reduce the computation time by decreasing the number of the input features, but also can ensure good classification accuracy. Therefore, it is important to discuss the classification accuracy for test 1 to test 4 under different contribution rates. Figure 7 shows the classification accuracy of micro-seismic events and blasts, total classification accuracy, and quality evaluation parameter MCC for test 1 to test 4 under different contribution rates.

The effects of the contribution rate can be analyzed by dividing it into three value intervals, which are [0.50, 0.90], [0.90, 0.95], and [0.95, 1.00], respectively. Firstly, it can be seen clearly that the total classification accuracy of the four tests takes an overall increasing trend when the contribution rate ranges from 0.50 to 0.90. Secondly, the total classification accuracy for test 1 to test 4 are relatively stable when the contribution rate ranging from 0.90 to 0.95. In addition, when the values of the contribution rate for test 1, test 2, test 3, and test 4 are equal to 0.92, 0.95, 0.90, and 0.90, the four tests reach their optimal total accuracy, which are 93.65%, 93%, 94.5%, and 93.45%, respectively. Then, the total accuracy for test 1 to test 4 begin to decline when the contribution rates exceed their optimal values. Thirdly, when the contribution rate ranges between 0.95 and 1.00, it can be seen that the total accuracy of the four tests show fluctuations, which decrease firstly and then increase slightly. Therefore, it can be determined that the optimal values of the contribution rate for test 1 to test 4 are distributed in the interval [0.90, 0.95].

Table 4 lists the computation time of test 1 to test 4 under different contribution rates, where the computation process includes the reading of waveform image databases, PCA procedure, establishment of the discrimination model, and prediction of classification results.

As shown in Table 4, the computation time increases gradually when the contribution rate increases from 0.90 to 0.95, while the computation time increases rapidly when the contribution rate reaches 1.00. Specifically, the average computation time of the four tests is 627.38 s when the contribution rate is equal to 1.00, which is approximately 2.15 times and 1.72 times as large as the average computation time when the values of the contribution rate are 0.90 and 0.95, respectively, indicating that the computation efficiency is seriously affected. Another disadvantage for the contribution rate reaching 1.00 is that numerous waveform image features with little classification effect are generated, which may even represent the noises in the complex underground mining environment. Hence, it can be inferred that the excellent classification results of micro-seismic events and blasts in underground mines cannot be obtained by simply increasing the value of the contribution rate.

Based on the above analysis, we can preliminarily determine that the optimal value of the contribution rate should be between 0.90 and 0.95. To further determine the optimal value of the contribution rate, a new variable named computation efficiency per accuracy (CEA) is introduced to quantitatively evaluate the effects of contribution rate on classification accuracy and computation efficiency, which is defined as

(12) $$\mathrm{CEA}=\frac{\mathrm{Computation}\begin{array}{c} \mathrm{time} \end{array}}{\mathrm{Total}\begin{array}{c} \begin{array}{c} \mathrm{accuracy} \end{array} \end{array}},$$

The contribution rate that corresponds to the minimum CEA is the optimal, because less computation time will be consumed for reaching the same total classification accuracy. Therefore, the values of CEA for test 1 to test 4 under the contribution rates ranging from 0.90 to 0.95 are solved, which are listed in Table 5. Evidently, the values of CEA for test 1 to test 4 are minimum when the contribution rate is equal to 0.90. It can be concluded that the optimal contribution rate is 0.90, which can simultaneously ensure excellent classification accuracy and computation efficiency.

### 4.2. Computation Efficiency

PCA is used to transform the original features into the new uncorrelated features with lower dimension. Therefore, the computation efficiency can be improved by PCA as the number of the input features is decreased according to the contribution rate. Table 4 lists the computation time of test 1 to test 4 under different contribution rates. It can be seen clearly that the computation time increases with the increase of the contribution rate, where the contribution rate of 1.00 means that the input features are the original features without dimension reduction. Additionally, for test 1 to test 4, the average computation time difference between *σ* = 0.90 and *σ* = 0.95 (∆*t*_1_), that between *σ* = 0.95 and *σ* = 1.00 (∆*t*_2_), and that between *σ* = 0.90 and *σ* = 1.00 (∆*t*_3_) are 72.31 s, 263.31 s, and 335.62 s, respectively. Evidently, ∆*t*_1_ is small while ∆*t*_2_ and ∆*t*_3_ are particularly large. Moreover, the average computation time corresponding to *σ* = 1.00 is approximately 2.15 times and 1.72 times as large as the that corresponding to σ = 0.90 and σ = 0.95, respectively, indicating that PCA can greatly improve the computation efficiency.

Based on the calculated results, we can estimate that the average total classification accuracy of 93% is reached through the proposed discriminant method, where the computation time of about 320 s (less than 6 min) is needed to correctly identify approximately 1860 micro-seismic events or blasts. It is evident that this computation efficiency is able to satisfy the requirements of data processing for seismicity analysis in underground mines. However, it will take approximately 1860 min (31 h) to finish the same workload through the manual discrimination if we assume that about 1 min is needed for an experienced analyst to discriminate a micro-seismic events (blast). Compared to the manual discrimination and the discrimination methods without PCA, it is demonstrated that the proposed method for discriminating micro-seismic events and blasts in underground mines can significantly improve the computation efficiency.

### 4.3. Further Applications

The proposed discriminant method is effective as long as the waveform image databases are established. For a seismic monitoring system that works normally, it is easy to collect sufficient data by a period of time (e.g., several weeks) to establish waveform image databases of micro-seismic events and blasts. As the PCA derived waveform image features are effective and the SVM algorithm performs well for binary classifications, the two databases containing at least 100 records (e.g., 50 micro-seismic events and 50 blasts) are acceptable to apply the proposed method. In addition, the waveform image databases can be updated by supplementing the correct classified micro-seismic events and blasts. Therefore, the proposed waveform image method can be effective in different underground mines with the establishment and update of waveform image databases.

To sum up, the advantages of the proposed discriminant method are prominent. Specifically, it is effective with high classification accuracy and is automatic with superior computation efficiency. In addition, the proposed method is demonstrated to be robust as there are not many differences between the results of four cross validations. Nevertheless, in the underground mining processes, the mining methods may be changed due to the increase of mining depth and the change of mining circumstances and conditions. Along with the continuous update of waveform image databases, it may be necessary to update the unified signal duration that determined before to generate a new value, in order to adapt to the new databases and guarantee the classification accuracy. This could be a limitation for the proposed method as the unified signal duration needs to be updated periodically and the time for updating it needs to be judged by professional mining technical staff.

## 5. Conclusions

Currently, the discrimination of micro-seismic events is a significant problem in underground mine seismicity. Focusing on the disadvantages of the discrimination methods using seismic source parameters and waveform spectrum analysis, a novel waveform image method was proposed. The waveform image databases of micro-seismic events and blasts were established by using the full waveform data collected from the Yongshaba underground mine in China. PCA was applied to extract the original features from the two waveform image databases, which could get rid of the similarities between micro-seismic events and blasts as well as retain the differences. Then, the original image features were transformed into new uncorrelated image features with quantitative importance and lower dimension through PCA, where the contribution rate was utilized to quantitatively determine the amount of initial information contained in the derived image features. Furthermore, the PCA derived waveform image features were coupled with SVM algorithm to establish discrimination models and perform the cross-validation tests. With the contribution rate of 0.95, results of four groups of cross validation show that the optimal values for the classification accuracy of micro-seismic events and blasts, total classification accuracy, and quality evaluation parameter MCC are 97.1%, 93.8%, 93.60%, and 0.8723, respectively. In addition, the effects of contribution rate on classification accuracy and computation efficiency were discussed quantitatively. The optimal contribution rate was determined to be 0.90. It is concluded that the proposed waveform image method for discriminating micro-seismic events is accurate and automatic, which can provide high-quality seismic data for seismicity analysis in underground mines. As for the future work, we intend to investigate the possibility for using the deep learning algorithms to replace the SVM algorithm to further improve classification accuracy and computation efficiency. In addition, it is interesting to explore the possibility of merging the magnitudes of micro-seismic events into the discrimination process, which can provide insights for the real-time identification of micro-seismic events with large magnitudes as well as prevent potential mining-induced seismic hazards.

## Figures and Tables

**Table 1:** Datasets for the four groups of cross validation.

Test	Training Sets	Test Sets
1	E1, B1	E2, B2
2	E2, B1	E1, B2
3	E1, B2	E2, B1
4	E2, B2	E1, B1

**Table 2:** Results of principle components for test 1 to test 4.

Item	Principle Components							*D*_orig_	*D*_redu_
	PC_1_	PC_2_	PC_3_	PC_4_	PC_5_	…	PC*_D_*_redu_		
**Test 1**									
Eigenvalues ×10^5^	5.04	3.66	1.70	1.58	0.81	…	0.0061	2000	1157
Importance %	9.20	6.68	3.10	2.89	1.48	**…**	0.01		
Cumulative %	9.20	15.88	18.98	21.87	23.35	**…**	95.00		
**Test 2**									
Eigenvalues ×10^5^	5.29	3.55	1.66	1.61	0.81	**…**	0.0061	2000	1166
Importance %	9.67	6.49	3.03	2.94	1.47	**…**	0.01		
Cumulative %	9.67	16.16	19.19	22.13	23.60	…	95.01		
**Test 3**									
Eigenvalues ×10^5^	4.36	3.61	1.67	1.45	0.77	**…**	0.0064	2000	1195
Importance %	7.73	6.39	2.97	2.57	1.37	**…**	0.01		
Cumulative %	7.73	14.12	17.09	19.66	21.03	**…**	95.00		
**Test 4**									
Eigenvalues ×10^5^	4.58	3.50	1.64	1.49	0.78	**…**	0.0064	2000	1205
Importance %	8.11	6.20	2.90	2.65	1.37	**…**	0.01		
Cumulative %	8.11	14.31	17.21	19.86	21.23	**…**	95.01		

**Table 3:** Classification results and quality evaluations for test 1 to test 4.

Test	TE	TB	FE	FB	Total Accuracy (%)	MCC
1	950	922	78	50	93.60%	0.8723
2	923	938	62	77	93.05%	0.8600
3	971	899	101	29	93.50%	0.8722
4	947	890	110	53	91.85%	0.8384

**Table 4:** Computation time (*s*) of test 1 to test 4 under different contribution rates.

Contribution Rate *σ*	Test 1	Test 2	Test 3	Test 4	Average
0.90	290.17	287.48	294.91	294.48	291.76
0.91	299.42	297.75	295.43	300.33	298.23
0.92	302.82	302.14	306.21	315.84	306.75
0.93	310.47	315.71	318.39	331.17	318.94
0.94	329.24	327.83	337.40	347.53	335.50
0.95	366.65	356.66	366.74	366.24	364.07
1.00	630.75	634.65	624.06	620.05	627.38

Note: The CPU model used in the four tests is Intel(R) Core (TM) i5-6500 CPU @ 3.20GHz.

**Table 5:** CEA for test 1 to test 4 under different contribution rates.

Contribution Rate *σ*	Test 1	Test 2	Test 3	Test 4
0.90	312.01	312.48	312.07	315.12
0.91	321.61	324.35	313.95	323.28
0.92	323.35	330.93	325.24	340.16
0.93	332.94	343.54	339.43	355.91
0.94	352.51	353.27	357.98	375.30
0.95	391.72	383.51	392.24	398.74
